# Management of a rare case of arrhythmogenic right ventricular dysplasia in pregnancy: a case report

**DOI:** 10.11604/pamj.2014.19.246.3773

**Published:** 2014-11-05

**Authors:** Jarraya Anouar, Smaoui Mohamed, Kolsi Kamel

**Affiliations:** 1 Anesthesiology Department, Hedi Chaker University hospital, Sfax, Tunisia

**Keywords:** Arrhythmogenic right ventricular dysplasia, cardiomyopathy, pregnency, anesthesia specificities

## Abstract

Arrhythmogenic right ventricular dysplasia is a rare but not exceptional inherited cardiomyopathy characterized by fibrofatty replacement of the myocardium of the right ventricular which could lead to serious arrhythmia and sudden death. Only a few cases of pregnancies with ARVD have been reported. The aim of this case presentation is to describe the clinical characteristics and anesthetics specificities in management of this disease in pregnancy and in delivery. We report the case of a young woman aged 28 years old with a past history of ARVD treated by medical treatment with radiofrequency ablation. This patient was pregnant and it was scheduled for cesarean section delivery. Preoperative evaluation showed a well tolerated pregnancy inspite of the severity of the ARVD. It was a severe form of ARVD because of RV dilation, the spread of the disease to LV and the history of ventricular tachycardia during an attempted of ablation by radio frequency. The treatment received by the patient was kept until the day of surgery. The act took place under general anesthesia. The postoperative period was uneventful and morphine titration was used for pain relief. The patient exits the hospital 3 days after delivery and breastfeeding was forbidden. We should pay attention on this disease witch is not yet well known and witch is highly risky in the peri-partum period. Even if pregnancy may be tolerated in moderate forms of ARVD, conception and delivery sould be discouraged especially in severe forms.

## Introduction

Arrhythmogenic right ventricular dysplasia (ARVD) is a rare hereditary cardiomyopathy characterized by a fibro-fatty replacement of right ventricular myocardium resulting in severe ventricular arrhythmias and life-threatening cardiac dysfunction [1–3]. The main symptom of the disease is ventricular arrhythmia witch can leads to palpitations, syncope or sudden death. In advanced stage of the disease, right or biventricular heart failure can be seen. The diagnosis of ARVD is based on a combination of clinical, morphological and electro cardiographic criteria detailed by the European Society of Cardiology (ESC) and the World Heart Federation [4]. The treatment includes the anti-arrhythmic drugs, the radiofrequency ablation, implantable defibrillator and surgery for advanced forms [5]. The prognosis of the disease remains difficult to establish but the identification of individual risk factors for sudden death, such as history of ventricular arrhythmias poorly tolerated or extension of the ventricular dysfunction, is fundamental to guide the therapeutic strategy. This risk seems to be reduced to known and treated forms [5]. Pregnancies with dilated and hypertrophic cardiomyopathies are common, but only a few cases of pregnancies with ARVD have been reported [6]. Pregnancies complicated by mild to moderate ARVD can be managed successfully but patients with end-stage ARVD should be discouraged from conceiving [6]. Therefore, it is difficult to assess the risks of pregnancy and delivery in patients with ARVD. The aim of this case report is to describe the clinical characteristics and anesthetics specificities of this disease in pregnancy. Here we report our experience of a pregnant patient with severe ARVD and we discuss the anesthesia management for cesarean section delivery and for the postpartum.

## Patient and observation

We report the case of a patient aged 28 years old with a personal past history of type II diabetes, dyslipidemia treated with diet alone, and family history of atrial fibrillation in the mother. The patient was followed for ARVD since 5 years ago when the patient suffered from the onset of palpitations related to ventricular arrhythmia. The diagnosis of ARVD was referred to the right ventricular dilation and reduced left ventricular ejection fraction estimated at 45% and frequent premature ventricular complexes in the stress test and late potentials in the high amplification Electocardiogram.

The patient was initially treated with Cordarone and because of the persistence of ventricular tachycardia and the occurrence of secondary hyperthyroidism the treatment was changed into flecainide. Then, in front of the recurrence of ventricular tachycardia, hyperten (β blocker) was introduced. Radiofrequency ablation was indicated later because of the failure of this medical treatment. A first attempt of radiofrequency treatment was complicated by ventricular tachycardia with ST elevation and troponin increase. Coronary angiography was normal. A second attempt of radiofrequency treatment was successfully made in the same year and the patient was treated by Detensiel, flecainide and Aspégic. Since the patient hasn't shown ventricular arrhythmias. Our patient was pregnant and pregnancy was well tolerated. During pregnancy, our patient didn't show ventricular arrhythmia. She was scheduled for cesarean section delivery at the 38th week of pregnancy.

Preoperative evaluation showed a pregnant woman in the 34 week of pregnancy with normal somatic examination. Cardiovascular examination showed sinusal cardiac rhythm. The ECG showed a right bundle branch block and inverted T waves in right precordial leads beyond V1. The chest radiography showed signs of right ventricular hypertrophy and a cardiothoracic index of 0.6. A transthoracic echocardiogram has been requested since and it has shown a dilated RV and reduced LVEF to 40%. Biological blood analyses (blood cell count, prothrombine ration and liver and kidney blood analyses) were correct We prescribed 6 hours of preoperative fasting and we continued the same treatment received by the patient (Detensiel, and Flucaine ASPEGIC) until the day of surgery. At the end of the consultation we explained the risk of bleeding, transfusion and the risk of per operative cardiac rhythm disorders. We informed the patient about the anesthetic techniques (general anesthesia) and postoperative analgesia by titration of morphine.

The day of surgery, we verified the checklist of the operatory room and we verified the presence of defibrillator. After the standard means of monitoring (ECG, pSO2, non invasive blood pressure and capnography), two peripheral venous lines of 18 G were taken. The patient was put in left lateral decubitus position. We prescripted 500 ml of Ringer lactate over 30 minutes. Three minutes before induction of anesthesia, the preoxygenation was performed. The anesthetic induction was done after the installation of drapes, in rapid sequence by propofol (3mg/kg), Remifentanil (1µg/kg) and Rocuronium (0.6 mg / kg) with Sellick maneuver. Intubation was carried out by a tube of 7Fr. The anesthetic maintenance was given by continuous infusion of propofol (10 mg / kg / hour) and remifentanil (0.1 mcg / Kg/ min). After fetal extraction, we administered a prophylactic antibiotherapy (2g of Cefazolin). Oxytocin was administered at a dose of 5UI intravenous injection, and 10 IU in 500 ml infusion of saline.

Intraoperative hemodynamic status was maintained stable with a systolic blood pressure of 110-100 mm Hg, diastolic blood pressure of 60-50 mm Hg and heart rate between 60 and 70 beats per minute. During the intervention, no desaturation was noted, end tidal p CO2 was maintained between 36 and 40 mm Hg and intrathoracic pressure did not exceed 25 mm Hg. Extubation was performed on the operating table. The postoperative period was uneventful with no electric changes on ECG and no bleeding. The postoperative analgesia was provided by paracetamol (15 mg/kg/6 hours) and morphine (5 mg subcutaneously every 6 hours). The patient leaved hospital 3 days after delivery and breastfeeding was proscribed.

## Discussion

In this observation, we reported the case of a pregnant woman with a severe form of ARVD, which is scheduled for cesarean section delivery. This is a rare situation with high risk of peri operative arrhythmia and mortality [7]. The incidence and frequency of occurrence of dysplasia with classical signs of the disease are estimated at 1 in 10 000 [7]. This incidence may be underestimated. In fact, there are a large number of clinical forms unrecognized and manifest by sudden death due to severe arrhythmias occurring as the first manifestation of the disease. This is nearly one third of cases by an American series of 100 cases [7]. Sudden death in ARVD affects athletes mainly. Corrado et al reported a series of 22 cases of sudden deaths in athletes with ARVD [8]. In addition, there are other forms of ARVD with congestive heart failure at the moment of the diagnosis and that may be considered as idiopathic dilated cardiomyopathy [9]. A family history of ARVD is present in 30% to 50% of cases [10, 11]. This implies that we should think to ARVD in case of family history of sudden death especially in the pre anesthetic consultation.

ARVD should be considered as a diagnosis in patients with symptomatic or asymptomatic VT (of left bundle branch block, LBBB, configuration) in the absence of apparent heart disease, especially if there is a family history. Post mortem, it should be considered a diagnosis in cases of sudden cardiac death particularly during exercise or peri-operatively, especially in young men. Proposed diagnostic criteria based on family history as well as structural, functional, and electrocardiographic abnormalities [12, 13]. Numerous diagnostic modalities assist in the diagnosis and evaluation of ARVD/C of which the ECG, echocardiography, angiography, radionuclide ventriculography, computed tomography and magnetic resonance imaging (MRI) are useful but depend in part on availability of local expertise. Emergency physicians and anaesthesiologists should at least be aware of common ECG patterns in ARVD0. About 50% of patients have an abnormal ECG at presentation [14] but within 6 years of diagnosis, virtually all patients will have one or more of the following findings during sinus rhythm: complete or incomplete right bundle branch block, QRS prolongation (in the absence of RBBB), epsilon wave in leads V1-V2, T-wave inversion in leads V1-V3, delayed (i.e. ‡ 55 ms) S-wave upstroke in leads V1-V30.

The role of pre-anesthetic consultation is not only to screen and diagnose ARVD but also to estimate the severity and starting up treatment preoperatively if possible. Severity criteria are essentially the RV dilation, the spread of the disease to the LV (left heart failure) and the occurrence of severe life-threatening arrhythmias [7]. Our patient was considered as severe as it has presented all these criteria of severity. Strategies for treatment appear to be based on local experience gained at the different centers. There are no specific guidelines for selecting patients to be treated with b-blockers, anti-arrhythmics, or radio frequency or implantable defibrillators [15]. When the disease has progressed to ventricular failure, treatment consists of the current therapy for heart failure, including diuretics, beta-blocking agents, angiotensin-converting enzyme inhibitors, and anticoagulants. In case of refractory heart failure and/or arrhythmias, cardiac transplantation may be the only remaining alternative [16].

During pregnancy, plasma volume, cardiac output and heart rate increase. The hematocrit fall and anemia may be physiological [17]. Tolerance of these physiological changes in women suffering from ARVD is not predictable. In the literature, cases of pregnancy in women with ARVD are very rare. Nilgün Güdücü et al [6] reported the case of a woman aged 26 years old suffering from ARVD who stopped her anti-arrhythmic treatment during pregnancy. This woman tolerated pregnancy to the end of 30 weeks of gestation. In an older series [18], patients with non severe forms can tolerate the hemodynamic changes of pregnancy with an increased risk of arrhythmias in the third quarter of pregnancy and peripartum. For our patient, although it is a severe form of ARVD, our patient did not show signs of intolerance of pregnancy. The best mode of delivery for parturiants suffering from ARVD is not yet well known [18]. For our patient, cesarean section was indicated because it was an advanced form of serious ARVD. In the series of Bauce et al [18], reporting 6 cases of ARVD with pregnancy, vaginal delivery was accepted in only 2 cases with epidural analgesia, whereas the remaining 4 cases were delivered by cesarean section under general anesthesia to prevent labor pain and hemodynamic changes of epidural analgesia.

We should focus on the intraoperative period because it is characterized by an increased risk of occurrence of severe rhythm disorders and sudden death. In a series of 50 cases of irreversible cardiac arrest during surgery, 36% of these cases had histologic evidence of right ventricular dysplasia at autopsy [19]. In the cesarean section, spinal anesthesia is the most common anesthetic technique [20]. It allowed a significant reduction in mortality [20]. However, for our patient, we opted for general anesthesia as it is a severe form of ARVD and to avoid hemodynamic changes following spinal anesthesia. Alexoudis AK et al [21] have detailed the anesthetic implications of ARVD and recommended general anesthesia for severe forms and have also recommended it to avoid high doses of bupivacaine in loco regional anesthesia for moderate forms. Propofol seems most used in case of ARVD for the induction of anesthesia [22] and the best suitable curare to our situation is rocuronium [21]. We added remifentanil in induction to prevent sympathetic stimulation following the laryngoscopy to provide better hemodynamic stability [20]. In postpartum period, analgesia is so important because pain can promote sympathic stimulation which can result in severe arrhythmia [8]. Breastfeeding can cause loss of electrolytes especially magnesium which may promote arrhythmias [23]. For our patient breastfeeding was proscribed not only for this reason but also because of the crossing of anti-arrhythmic drugs in breast milk.

## Conclusion

Knowledge of this disease has been improved significantly over the last decade following the creation of an international registry. However, there are no guidelines in the therapeutic management of these patients and the treatment is based mainly on the experience of each team. In anesthesiology, the preoperative consultation is a good opportunity for screening this disease in order to take precautions. This would avoid the occurrence of arrhythmias or serious irreversible cardiac arrest immediately at anesthetic induction or even postoperatively. Despite that there are several case reports of ARVD in pregnancy, showing the experience of different anesthesia teams, there are no specific recommendations in terms of technical or anesthetic protocols.
